# Resting frontal alpha asymmetry as a predictor of executive and affective functioning in children with neurodevelopmental differences

**DOI:** 10.3389/fpsyg.2022.1065598

**Published:** 2023-01-13

**Authors:** Sarah R. Edmunds, Jason Fogler, Yael Braverman, Rachel Gilbert, Susan Faja

**Affiliations:** ^1^Department of Psychology, University of South Carolina, Columbia, SC, United States; ^2^Division of Developmental Medicine, Boston Children’s Hospital, Boston, MA, United States; ^3^Departments of Pediatrics & Psychiatry and Behavioral Sciences, Harvard Medical School, Boston, MA, United States; ^4^Leadership Education in Neurodevelopmental & Related Disabilities/Institute for Community Inclusion, Boston, MA, United States; ^5^Department of Pediatrics, Johns Hopkins University, Baltimore, MD, United States

**Keywords:** autism, executive function, emotion regulation, EEG, asymmetry, ADHD, neurodevelopment

## Abstract

The relative difference of resting EEG frontal alpha activation between left and right hemispheres (FAA; i.e., asymmetry) correlates with global approach and avoidance tendencies. FAA may relate to problems with executive and affective functioning in children with neurodevelopmental differences, including autism and ADHD. We (1) characterize relative left vs. right FAA in autistic, ADHD, and neurotypical children (NT) and (2) investigate whether FAA predicts “hot” executive function or emotion dysregulation. Participants were 97 7- to 11-year-old autistic, ADHD, and NT Children. Children with ADHD displayed greater left (relative to right) FAA compared to autistic and neurotypical children. Children with ADHD displayed greater challenges with “hot” EF on a gambling task than autistic children, whereas children with co-occurring autism and ADHD had greater parent-reported emotion dysregulation than NT and autism-only groups. Greater left FAA predicted worse hot EF for all children but was not significantly related to emotion dysregulation. Regardless of clinical diagnosis, relatively greater left FAA relates to hot EF. While hot EF deficits may be specific to ADHD rather than autism, both together confer additive risk for emotion dysregulation. Future research should explore the functional relation between FAA, reward processing, and affect for children with different EF-related neurodevelopmental differences.

## Introduction

Identifying early childhood risk factors of later functional impairment can inform prevention intervention efforts during children’s early years, which represent a period of increased neuroplasticity (Wakschlag et al., 2019). Increasingly, the National Institute of Mental Health’s Research Domain Criteria (RDoC) perspective encourages a focus on dimensional risk that spans traditional diagnostic categories, can be measured at multiple levels, and explains functional impairment (Insel et al., 2010). For example, while one might characterize neurodevelopmental disorders as discrete diagnostic categories [i.e., attention-deficit/hyperactivity disorder (ADHD); autism spectrum disorder (ASD)], children with neurodevelopmental disorders may be more *dimensionally* characterized as having challenges with impulsivity, hyperactivity, social interaction, and/or emotion dysregulation.

Emotional dysregulation is a powerful predictor of mental-health-related functional impairment (e.g., Mazefsky et al., 2013; Graziano and Garcia, 2016). Emotional regulation is the ability to change or calm one’s emotional state and involves the dual development of both bottom-up approach/avoidance reactivity and top-down executive control (Zelazo and Carlson, 2012; Grabell et al., 2017; Wakschlag et al., 2018). Some emotion regulation ability may be inexorably linked to trait-based emotion reactivity, whereas other emotion strategies are cognitive in nature and are more malleable (Jahromi et al., 2012). Both ADHD and ASD are associated with increased emotion regulation challenges (Shaw et al., 2014; Conner et al., 2021; Day et al., 2022), but the mechanisms by which this is the case are unclear. In ADHD, reduced top-down executive control may contribute to increased emotion dysregulation (Shaw et al., 2014). In ASD, decreased emotion regulation may mechanistically related to ASD characteristics in part due to reduced brain activation during cognitive appraisal of emotive faces (Richey et al., 2015).

Frontal alpha EEG asymmetry is one long-studied physiological index of emotional reactivity within the RDoC’s Arousal and Regulatory Systems domain (Wheeler et al., 1993; Coan and Allen, 2004; Gatzke-Kopp et al., 2014). Brain activity in the alpha frequency band reflects individual differences in awareness and attention (Hanslmayr et al., 2011), and may play a role in both emotion reactivity and regulation (Wheeler et al., 1993). Frontal alpha asymmetry (FAA) is operationalized as the relative difference in frontal alpha activation between left and right hemispheres and is thought to reflect approach (left) and avoidance (right) impulses. FAA measured during a resting state is viewed as a temperament or “trait” measure of approach/avoidance emotional reactivity. Resting FAA predicts clinically significant individual differences in internalizing and externalizing symptomatology in children (Baving et al., 2002; Blackhart et al., 2006; Gatzke-Kopp et al., 2014).

However, FAA may more accurately index the top-down executive control aspect of emotion regulation than bottom-up emotion reactivity. The asymmetric inhibition model (Grimshaw and Carmel, 2014) explains the relation between FAA and the executive function-dependent aspects of emotion regulation. They cite EEG studies using source localization procedures to highlight that FAA may most directly reflect the activity of the dorsolateral prefrontal cortex (dl-PFC). The dl-PFC is centrally involved in executive functioning, including behavioral inhibition, and is active during the cognitive appraisal of emotion. According to the model, the left dl-PFC acts to inhibit avoidance-based motivational urges as they distract from one’s intentional goals, while the right dl-PFC acts to inhibit approach-based motivation as they distract from one’s intentional goals. In this manner, relatively lower left (and therefore, higher right) FAA reflects poorer executive control of internalizing emotions like sadness and anxiety, while relatively lower right (and therefore, higher left) FAA reflects poorer executive control of externalizing, approach-oriented emotions like anger and joy. There is ample literature to reflect this left-externalizing vs. right-internalizing FAA pattern (see Grimshaw and Carmel, 2014 for a review).

FAA may contribute differentially to cascading clinically impairing challenges with affective regulation in children with different neurodevelopmental disorders. At least 50% of autistic children and 33% of children with ADHD present with co-occurrence of anxiety and depression, including 15–20% with a mood disorder by preschool or elementary school (Salazar et al., 2015; Schendel et al., 2016). Executive function (EF) deficits are highly linked to the behavioral regulation of emotion in both ASD and in all children (Demetriou et al., 2019). EF is a core deficit in ADHD (Seidman, 2006) and linked to emotion dysregulation in these children (Garcia et al., 2020). Autistic children also have less developed executive function (Demetriou et al., 2019), including delay of gratification (Faja and Dawson, 2013), a skill that involves the top-down inhibition of a prepotent response in the “hot” or emotionally valenced context of reward.

There is clear evidence of diagnostic group differences in resting FAA for ADHD, but findings are mixed for autism. Children with ADHD demonstrated greater relative left FAA during a behavioral inhibition task than did NT children (Ellis et al., 2017), which suggests that they have higher approach motivation or less inhibited avoidance motivation than NT children. Similarly, adults with ADHD have greater relative left FAA than NT adults (e.g., Keune et al., 2015). In autism, a study of 6- to 18-month-olds at higher likelihood for ASD by virtue of having an older sibling with ASD found that these infants had relatively greater right FAA than “low likelihood” infants at 6 months, but that this pattern faded by 12 months (Gabard-Durnam et al., 2015). Children aged 9–14 years with ASD and no intellectual impairment were found to have relatively greater left midfrontal activation than typically developing children (Sutton et al., 2005). A similar study of 8- to 15-year-old children with ASD and below average to superior verbal IQ found no diagnostic group differences in the number of children with relative left versus right FAA, measured dichotomously (Burnette et al., 2011). To summarize, group differences in FAA are complicated by measurement (i.e., dichotomous vs. continuous asymmetry; lateral vs. midfrontal) and cognitive ability. Inconsistent findings also potentially strengthen the hypothesis that FAA may better reflect transdiagnostic characteristics than diagnostic categories.

It appears that FAA predicts emotion dysregulation for children in general, but it is unclear whether diagnostic group (autism, ADHD) moderates the strength of this relationship. For all children, emerging evidence supports the relation between relatively greater resting right FAA and broad measurements of later internalizing symptoms (Diego et al., 2006; Smith and Bell, 2010) as well as relatively greater resting left FAA and later externalizing symptoms (Smith and Bell, 2010). There is mixed evidence as to whether the relation between FAA and emotion dysregulation differs for autistic children compared to neurotypical (NT) children or children with ADHD. In a study of autistic adolescents, Schiltz et al. (2018) found that individuals with relatively greater right FAA had greater anxiety symptoms, which is consistent with previous literature on FAA and anxiety in the general population. However, in the two studies of FAA in slightly younger children with ASD and NT development, the relation between asymmetry and internalizing symptoms in ASD is less clear. Specifically, for their ASD samples, both studies found that increased relative *left* FAA was correlated with greater general anxiety (Sutton et al., 2005) and higher OCD and anger-related symptoms (Burnette et al., 2011), which is opposite what would be expected. Finally, while left FAA may predict behavioral inhibition in ADHD (Ellis et al., 2017), there has been little research on whether FAA predicts EF in emotionally valenced contexts (i.e., “hot” EF) in autism.

### The current study

Understanding diagnostic group and transdiagnostic differences in FAA may help the field better understand heterogeneity in autism as well as co-occurring mental-health challenges (Schiltz et al., 2018). If FAA either differs by diagnostic classification or predicts individual differences in executive functioning or emotion dysregulation, it would represent a risk factor that is easily measurable at an early age, may identify subgroups of children, and may predict differential responses to intervention. The goals of this study were to:

1. Characterize relative left vs. right frontal alpha activation (FAA) in children with autism, ADHD, co-occurring autism and ADHD, and neurotypical development (NT). Specifically, we hypothesized that children with ADHD will show greater relative left FAA than other children. We did not make a directional hypothesis about FAA in autistic children because previous findings are mixed.
2. Investigate the extent to which children’s FAA predicts “hot” EF during a behavioral task. Because hot EF tasks involve approach/avoidance decision-making within an emotionally valenced environment, we predicted that FAA will be related to hot EF for all children regardless of diagnosis.
3. Relate children’s FAA to parent-report measures of their emotion dysregulation. We predicted that FAA will be related to emotion dysregulation, given prior work. We hypothesized that this relation will be stronger for children with ADHD than autism, given the more consistent evidence of a relation between FAA and mental-health challenges in ADHD compared to autism.

## Materials and methods

### Participants

Participants were 97 7- to 11-year-old children with autism spectrum disorder (ASD; *n* = 29), attention-deficit/hyperactivity disorder (ADHD; *n* = 27), co-occurring autism and ADHD (*n* = 16), and neurotypical development (NT; *n* = 25). Participants were 8.30–8.93 years old on average, 74–93% male (*n* = 82), and 67–84% White (depending on group). Demographic characteristics are reported in Supplementary Table 1. Participants were recruited through a research registry, clinical referrals, community sources, and word of mouth. Exclusion criteria included colorblindness, inability to complete procedures in English, below-average cognitive ability, sensory or motor impairments that impeded ability to complete the test battery, medical disorders or medications that impact the central nervous system, history of seizures or use of seizure medication, and prolonged prenatal substance exposure. Other medication use (stimulant and non-stimulant) was non-exclusionary and did not differ by group (see Faja et al., 2021). The study was conducted at a hospital in New England and approved by its Human Subjects Division. All parents provided written consent and children provided written assent to participate.

This sample size allows for the detection of medium to large effect sizes with the ANCOVA (one covariate) and regression approaches planned for this study. Prior literature supports the expectation of medium to large effect sizes (e.g., Sutton et al., 2005; Ellis et al., 2017).

### Procedure

Parents of participants completed a phone screening to establish eligibility. Diagnostic status and cognitive ability were assessed during the first visit, and findings were supervised and reviewed by a licensed psychologist. Over two additional visits, all participants completed a battery of executive function (EF) and social cognition tasks while parents completed questionnaires about children’s behavior and functioning. A subset of participants completed additional visits for an intervention study; only baseline data are considered here.

### Measures

Participants in the present study completed clinical characterization, EEG resting data collection, and the Hungry Donkey gambling task (see below). Participants’ parents then completed a questionnaire about their children’s behavioral and emotional functioning.

#### Clinical characterization

All participants completed assessments of cognitive and adaptive functioning using the Vineland Adaptive Behavior Scales, Second edition (Vineland-2; Sparrow et al., 2005), and the Wechsler Abbreviated Scale of Intelligence-2 (WASI-2; Wechsler, 2011), which also assured verbal ability.

All children in the ASD group had an existing diagnosis of ASD. Diagnosis was confirmed according to DSM-5 (American Psychiatric Association, 2013) criteria based on expert clinical judgment, the Autism Diagnostic Interview-Revised (ADI-R; Rutter et al., 2003), and the Autism Diagnostic Observation Schedule, second edition (ADOS-2; Lord et al., 2012). ADHD symptoms were assessed as a continuous variable, using the ADHD subscale of the Child Behavior Checklist (CBCL; Achenbach and Rescorla, 2001). There is little agreement on how to quantify ADHD symptoms in autistic children; as such, a T-score of 65 was used as a cut-point to identify children within a borderline clinical range of ADHD for the ADHD group (Achenbach and Rescorla, 2001), in line with other work (Andersen et al., 2013; Cremone-Caira et al., 2021). Children with a confirmed autism diagnosis and T-scores ≥65 on the CBCL ADHD subscale were determined to have clinically significant ADHD symptoms and were included in the ASD + ADHD group.

#### Resting EEG data collection

Participants completed 2 minutes of alternating eyes open, eyes closed resting EEG data collection. During eyes open periods, children were asked to fixate on a central cross on a screen directly in front of them. Neural responses were continuously recorded *via* a Net Amps 400 (Electrical Geodesics, Inc.) using the 128-channel HydroCel sensor net 2.0 (HSN).

#### Resting EEG data preprocessing

EEG data preprocessing and power analyses were conducted using the Batch EEG Automated Processing Platform (BEAPP; Levin et al., 2018). Data were first bandpass filtered using a high pass (1 Hz) and low pass (100 Hz) filter and then downsampled from 500 to 250 Hz.

Next, data were artifact detected and corrected using the Harvard Automated Preprocessing Pipeline for EEG (HAPPE), a pipeline optimized for short recording EEG data collected from young children with neurodevelopmental disorders (Gabard-Durnam et al., 2018). HAPPE first applied linenoise removal to the data at 60 Hz, and next performed detection and removal of bad channels. Artifacts in the data, such as eyeblinks and eye or muscle movements, were automatically detected using first wavelet-enhanced independent component analysis (w-ICA) and then ICA with the multiple artifact rejection algorithm (MARA; Winkler et al., 2011, 2014). The following channels, in addition to the 10–20 electrodes, were used for ICA with MARA: 34, 28, 16, 1, 47, 51, 37, 60, 72, 30, 6, 117, 105, 116, 32, 98, 97, 85, 87, and 75. Electrodes were spread evenly across the scalp, and the number of electrodes was chosen relative to our recording length to maximize ICA performance and prevent overfitting of the algorithm (Särelä and Vigário, 2003). Following artifact removal, bad channels were interpolated and each channel was re-referenced to the average of all channels.

Data were then segmented into 2 s windows. Segments were inspected again for artifact and segments with an amplitude greater than 40 μV were not included in the final data analysis. A 40 μV cutoff reflects the smaller amplitude that results from the wavelet thresholding and ICA steps during artifact detection (Gabard-Durnam et al., 2018).

#### EEG power analysis

Using a fast Fourier transform with a 1 s Hanning Window, we computed the power spectrum for each segment for all electrodes: For each electrode, power across all segments was averaged for the alpha frequency band (8–13 Hz). Data within the alpha frequency band (8–13 Hz) from electrodes indexing left and right frontal activation (F3 and F4) were filtered and processed using BEAPP (Levin et al., 2018).

##### Left and right relative activation

The alpha frequency band (8–13 Hz) from electrodes indexing left and right frontal activation (F3 and F4) was extracted. F3 and F4 were selected to align with the convention in this literature, which allows for more direct comparison (Coan and Allen, 2004; Sutton et al., 2005; Reznik and Allen, 2018). Absolute power (mean power per hertz) was derived. Alpha power reflects the inverse of activation. Asymmetry in children’s FAA was calculated using residual values (Coan and Allen, 2004; Reznik and Allen, 2018). Residual values are more sensitive than traditional difference scores (Meyer et al., 2017). Left residual power values were residual values from a model of right power predicting left power (i.e., left power, controlling for right). Right residual power values were residual values from a model of right power, controlling for left. The inverse of each value was taken (i.e., the inverse of power is activation). Each variable (right and left residual) therefore represents unique frontal hemispheric activation. No significant difference in FAA by gender was found, *p* = 0.92.

#### Hungry donkey task: “Hot” executive function

The Hungry Donkey gambling task (Supplementary Figure 2) assesses children’s strategic long-term decision-making in the heightened emotional context of reward and is conceptualized here as a “hot” executive function (EF) measure (Crone and van der Molen, 2004). Children fed a cartoon “hungry” donkey by selecting freely from one of four doors across a total of five blocks, with 20 trials per block. Two doors were advantageous and resulted in a net gain (lesser vs. greater) of apples. Two doors were disadvantageous and resulted in a net loss (lesser vs. greater) of apples. This was therefore a reward contingency learning task that pitted immediate rewards against a net reward over time (on advantageous doors).

Contingency reward learning was operationalized as the ratio of advantageous to disadvantageous decisions made across the 20 trials of the final block (once children had a chance to learn door contingencies). For example, a score of −20 would indicate that a child made all disadvantageous decisions for the final block.

#### Parent questionnaire of emotion dysregulation

Participants’ caregivers completed the Child Behavior Checklist (CBCL; Achenbach and Rescorla, 2001), from which the 18-item Emotion Dysregulation Index (EDI) was derived (Samson et al., 2014). The CBCL is a normed parent-report questionnaire that assesses behavioral and emotional functioning. The EDI is an 18-item scale derived from CBCL items across subscales that indexes transdiagnostic emotion dysregulation and has been validated with autistic children (Samson et al., 2014; Berkovits et al., 2017).

## Results

### Relative frontal alpha activation differs by diagnostic group

**Figure 1:**
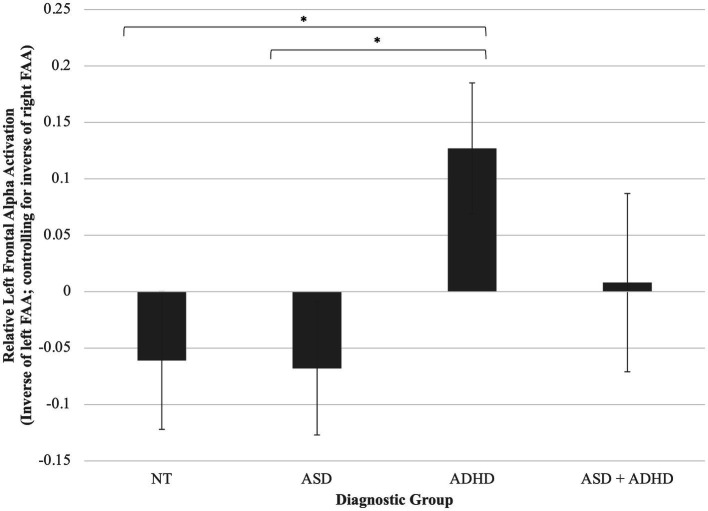
Group differences in left frontal alpha activation (relative to right). Children with ADHD displayed greater left (relative to right) FAA compared to both autistic, *p* = 0.020, and neurotypical children, *p* = 0.022, but not children with ASD + ADHD, *p* = 0.234. *p < 0.05. Error bars are +/− one standard error.

A one-way ANCOVA was conducted and found a trend toward a statistically significant difference between diagnostic groups on FAA, controlling for WASI-2 IQ, *F*(3, 96) = 2.45, *p =* 0.057, partial *η*^2^ = 0.07. Planned least significant difference (LSD)-corrected *post-hoc* pairwise comparisons of adjusted means revealed that children with ADHD displayed greater left (relative to right) FAA compared to both autistic, *p* = 0.020, and neurotypical children, *p* = 0.022, but not children with ASD + ADHD, *p* = 0.234 (Figure 1).

### FAA predicts “hot” executive function

**Figure 2:**
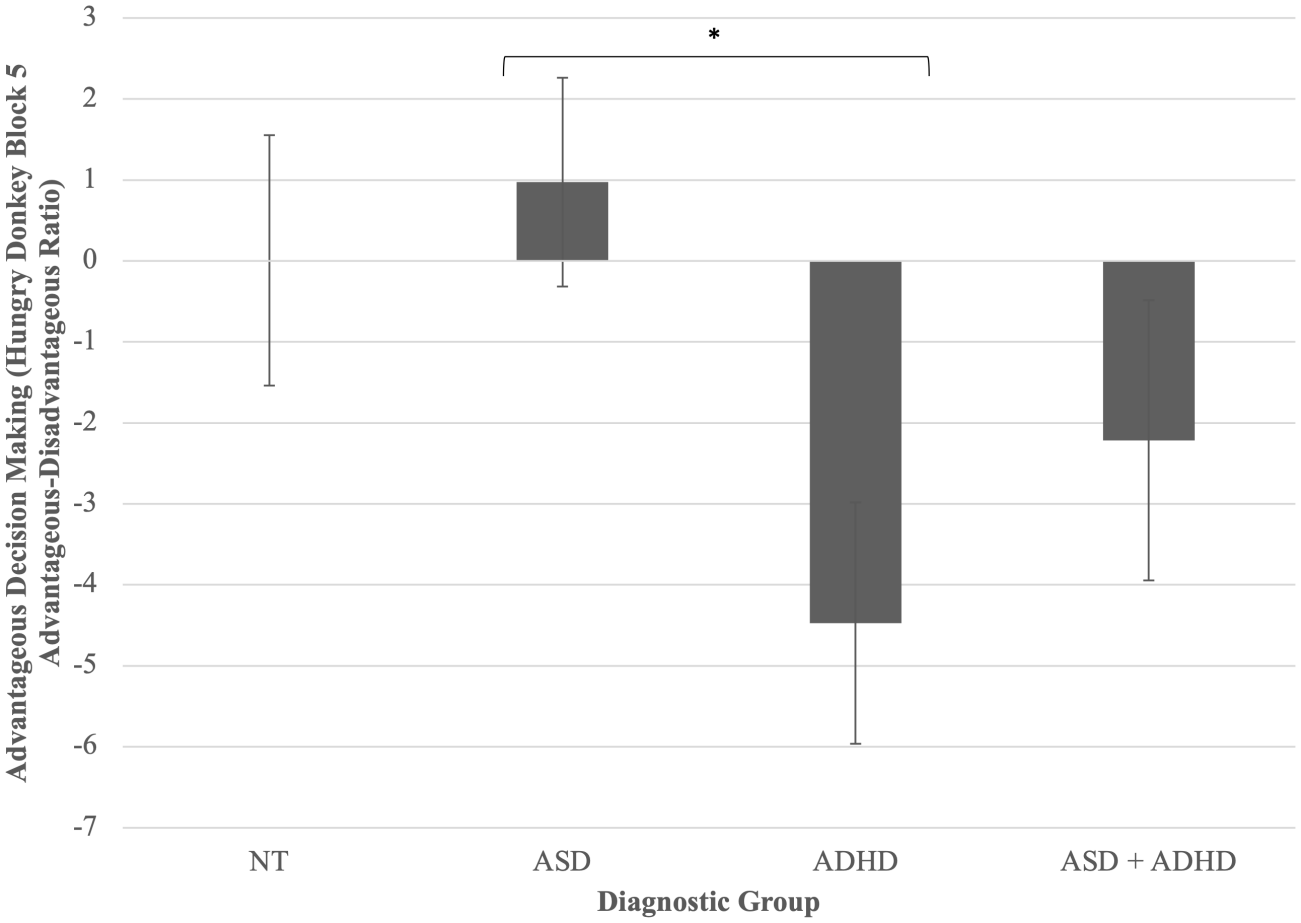
Group differences in reward contingency learning on the hungry donkey task. Children with ADHD displayed greater challenges with “hot” EF than did autistic children, *p* = 0.009. *p < 0.05. Error bars are +/− one standard error.

A one-way ANCOVA found a statistically significant difference between diagnostic groups on contingency reward learning during the Hungry Donkey task, controlling for WASI-2 IQ, *F*(3, 82) = 2.97, *p =* 0.037, partial *η*^2^ = 0.10. *Post-hoc* pairwise comparisons of adjusted means revealed that children with ADHD displayed greater challenges with “hot” EF than did autistic children, *p* = 0.009 (Figure 2). Other *post-hoc* comparisons were non-significant.

**Figure 3:**
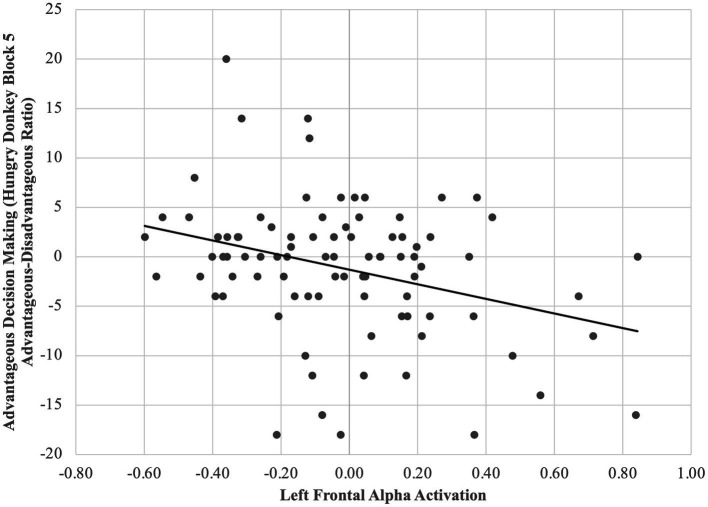
Higher relative left (vs. right) FAA predicts less advantageous decision-making across diagnostic groups. For all children, higher relative left FAA predicted worse reward contingency learning, *β* = 0.32, *p* = 0.002.

A multiple regression model controlling for IQ revealed that for all children, higher relative left FAA predicted worse reward contingency learning, *β = 0*.32, *p* = 0.002 (Figure 3). Moderation by group was non-significant, *p*s > 0.05.

### FAA does not significantly predict emotion dysregulation

**Figure 4:**
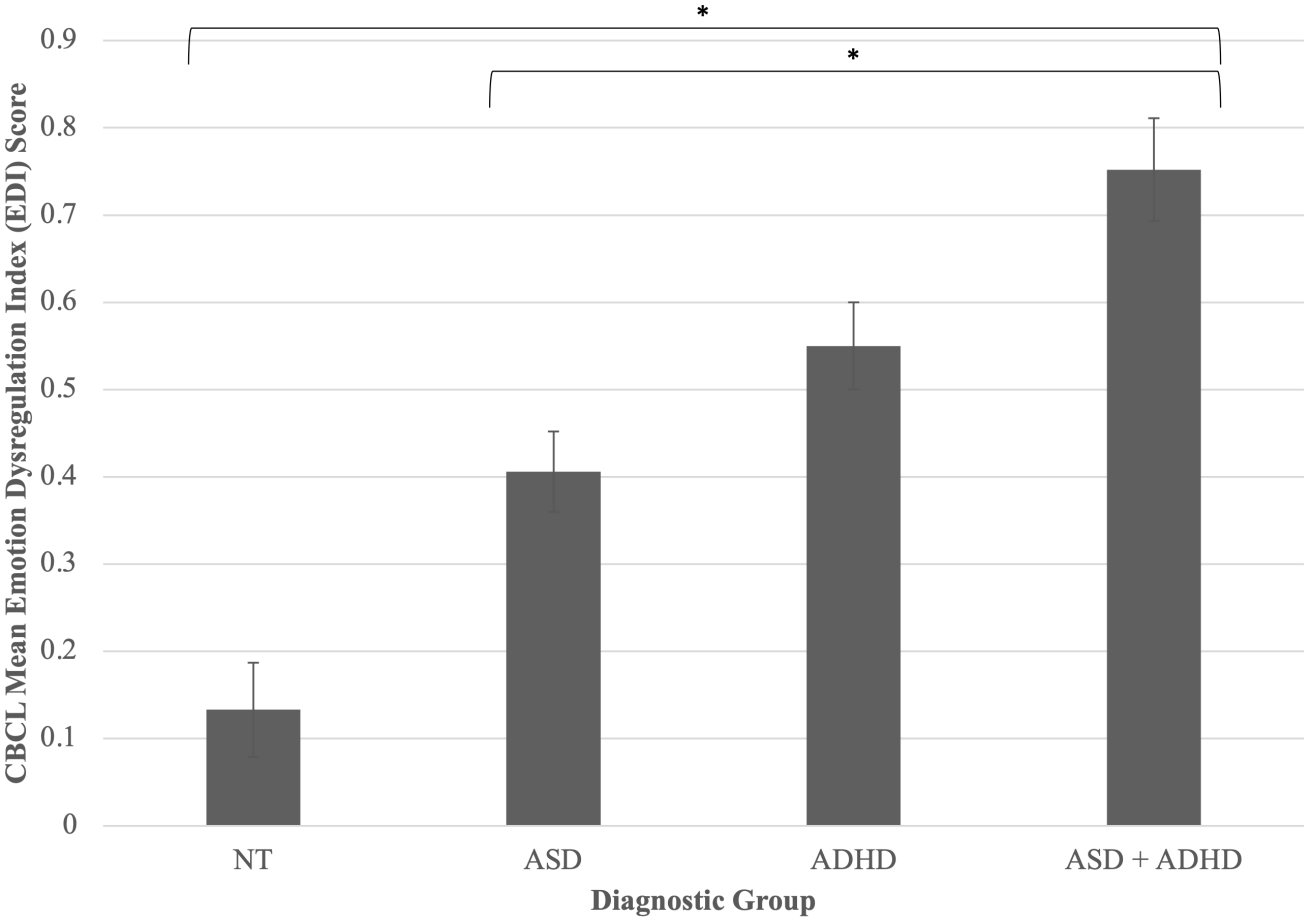
Diagnostic group differences in emotion dysregulation. Children with co-occurring autism and ADHD had greater caregiver-reported emotion dysregulation than NT children, *p* = <0.001, and children with autism alone *p* < 0.001. FAA was not significantly predictive of emotion dysregulation. *p < 0.05. Error bars are +/− one standard error. Difference between ASD + ADHD and ADHD groups trends toward significance (*p* = 0.06).

A one-way ANCOVA revealed a statistically significant difference between diagnostic groups on CBCL Emotion Dysregulation Index (EDI) score, *F*(3, 92) = 11.91, *p <* 0.001, partial *η*^2^ = 0.28. Of note, an additional 40 children who had caregiver-report data only were available for this analysis; results do not change when including these participants [*F*(3, 136) = 21.86, *p <* 0.001, partial *η*^2^ = 0.33; NT *n* = 33; ASD *n* = 46; ADHD *n* = 36; ASD + ADHD *n* = 26]. *Post-hoc* pairwise comparisons of adjusted means indicated that children with co-occurring autism and ADHD had greater caregiver-reported emotion dysregulation than NT children, *p* = <0.001, and children with autism alone *p* < 0.001 (Figure 4).

A multiple regression model controlling for IQ revealed that for the full sample, FAA was not significantly related to parent-reported emotion regulation, *β* = 0.15, *p* = 0.11. Moderation by diagnostic group was also non-significant, *p*s > 0.05.

## Discussion

Children with ADHD displayed greater left (relative to right) frontal alpha activation (FAA) compared to both autistic and neurotypical children. While children with ADHD displayed greater challenges with “hot” EF (reward contingency learning) than did autistic children, children with co-occurring autism and ADHD were reported to have greater emotion regulation challenges than neurotypical (NT) children and children with autism alone. Higher relative left FAA predicted worse reward contingency learning for all children, but it was not significantly related to parent-reported emotion dysregulation. The strength of these relations did not differ by diagnostic group.

Our hypothesis that children with ADHD would display greater left (relative to right) FAA compared to both autistic and neurotypical children was supported and is consistent with the literature (e.g., Keune et al., 2015; Ellis et al., 2017). However, we did not find an omnibus effect or any differences for autistic individuals compared to other groups. There is additional evidence that neurodevelopmental group differences in FAA may be moderated by both participant characteristics and task context. For example, researchers found a gender difference such that 4- to 8-year-old males with ADHD had relatively greater resting left FAA than NT males, while females with ADHD had relatively greater right FAA than NT females (Baving et al., 1999). Compared to NT children, 7- to 14-year-old children with ADHD had relatively greater left FAA during failed trials of a go/no-go task, a measure of inhibition, but not during other trials, although FAA was not measured at rest (Ellis et al., 2017).

We found that children’s relative left FAA was related to their executive function in a “hot,” emotionally valenced context. We also found that reward contingency learning scores during the Hungry Donkey task were worse for children with ADHD compared to all other groups, suggesting that this type of hot EF may be an ADHD-specific deficit. However, the relation between greater relative left FAA and hot EF performance held across all children. In other words, relatively greater approach motivation (or a lack of adaptive inhibition to avoidance) relates to reward contingency learning, regardless of neurodevelopmental diagnosis. This suggests that children who are better at inhibiting the extent to which their emotional reactivity affects their behavior may make better long-term decisions “under pressure.” That this finding is not specific to—or stronger for—children with ADHD, but rather extends to all children, suggests one of two things: that the effect of EEG-indexed emotional reactivity on in-the-moment EF behaviors is transdiagnostic, or that its effect is stronger and more detectable in relation to behavioral tasks than to more global or parent-report measures. For example, He et al. (2010) found that infants’ relative left FAA (i.e., increased approach motivation) was associated with increased anger *via* a behavioral observation measure (rather than parent-report measure). This is one of the few studies in children to demonstrate a link between frontal asymmetry and behaviorally measured action urges in emotionally valenced situations.

We observed differing levels of emotion dysregulation by diagnostic group, but FAA appears to be unrelated to emotion dysregulation in our sample. In contrast, Richey et al. (2015) found that for adults with ASD, activation in the dl-PFC (the same area that is theorized to be indexed by EEG FAA) indexed *via* fMRI was reduced when viewing emotive faces, compared to neurotypical adults. Therefore, while we found evidence for a neural mechanism (i.e., FAA) by which approach motivation may be related to reward contingency learning, we do not find a neural mechanism for emotion dysregulation. We also do not find group differences in FAA levels or the relation between FAA and our affective variables of interest, suggesting that FAA-based neural mechanisms may be transdiagnostic.

Children with co-occurring autism and ADHD had the highest reported challenges with emotion regulation, pointing toward an additive effect of neurodevelopmental differences on outward signs of emotion regulation. This kind of additive effect has rarely been observed (England-Mason, 2020). That FAA does not significantly predict or underpin these diagnostic differences in emotion dysregulation may point toward diagnosis-specific functional pathways for emotion dysregulation. For example, individuals with ASD but relatively low symptom severity may have increased internalizing or externalizing symptomatology as a result of the effort needed to “compensate” for social communication challenges inherent to the disorder (Livingston et al., 2019). While for children with ADHD, emotional reactivity may be more directly linked with impulsivity-related EF deficits, and some researchers even posit an “irritable subtype” of ADHD, for which emotion regulation challenges are central (Karalunas et al., 2019). More research is needed to explore these possibilities.

### Limitations and future directions

Limitations of this study include its cross-sectional design, lack of measurement of other potential covariates beyond IQ (e.g., social communication, baseline negative affect, cold EF), relatively small sample size, and reliance on caregiver report for emotion dysregulation and ADHD classification. While FAA, as a brain-based measure, is conceptualized as a more proximal influence on EF and emotion regulation than behavioral indexes of these variables, this study could draw stronger conclusions if children were studied longitudinally and at younger ages, when EF and emotion regulation first develop. To better understand the relation between FAA, reward processing, and affective functioning, future work should seek to understand whether the *structure or strength* of these processes fundamentally differs for children with different EF-related neurodevelopmental differences. For example, this study did not include a measure of EF outside of an emotionally valenced context (i.e., “cold” EF), or examine whether, as in adolescents with ADHD, negative affect moderates the degree to which FAA predicts diagnostic characteristics (Alperin et al., 2019). Future research should explore both “hot” and “cold” EF skills in autistic children and children with co-occurring ADHD.

This study does not include a measure of social communication, another important potential correlate of functional impairment in autism and ADHD. There have been mixed findings as to whether FAA relates to social impairment, and if so, whether the relation is mediated by emotion regulation challenges. For example, while Sutton et al. (2005) found that higher right FAA related to greater social communication challenges, Burnette et al. (2011) found that higher right FAA related to *fewer* restricted and repetitive behaviors. These findings are complicated by differences in the operationalization of FAA. More work is needed to understand how and if FAA may relate to social communication in children with neurodevelopmental disorders.

## Conclusion

This study was one of the first to examine whether children’s relative left or right frontal alpha activation (FAA) was related to their executive function (EF) in a “hot,” emotionally valenced context. We found that for all children, greater relative left FAA, or more approach-oriented motivation, related to worse functioning during a hot EF task. While there has been much more research linking FAA to internalizing and externalizing symptoms, we did not find a relation between FAA and emotion dysregulation-based symptoms in our sample, for all children or for children with ADHD or ASD specifically. Instead of comparing children by diagnostic category, commonly observed variability in functional impairment in children with neurodevelopmental disorders may be better explained by using an RDoC approach to explore dimensional characteristics in approach/avoidance reactivity, executive function, and clinically impairing emotion dysregulation.

## Data availability statement

The datasets presented in this study can be found in online repositories. The names of the repository/repositories and accession number(s) can be found at: The dataset analyzed for this study can be found in the National Database for Autism Research (NDAR reference number 2030).

## Ethics statement

The studies involving human participants were reviewed and approved by Boston Children’s Hospital IRB and University of Washington IRB. Written informed consent to participate in this study was provided by the participants’ legal guardian/next of kin.

## Author contributions

SE conceptualized the study, conducted the statistical analyses, and drafted the manuscript. JF and SF assisted in conceptualizing the study and reviewed intermediate and final drafts of the manuscript. YB processed the EEG data, drafted the EEG methods, and reviewed the final manuscript. RG collected behavioral and EEG data, conducted preliminary analyses, and reviewed the final manuscript. All authors contributed to the article and approved the submitted version.

## Funding

This study was supported by NICHD R00HD071966 to SF.

## Conflict of interest

The authors declare that the research was conducted in the absence of any commercial or financial relationships that could be construed as a potential conflict of interest.

## Publisher’s note

All claims expressed in this article are solely those of the authors and do not necessarily represent those of their affiliated organizations, or those of the publisher, the editors and the reviewers. Any product that may be evaluated in this article, or claim that may be made by its manufacturer, is not guaranteed or endorsed by the publisher.
