# Supplementary material for: Resting frontal alpha asymmetry as a predictor of executive and affective functioning in children with neurodevelopmental differences
Source: Front Psychol. 2023 Jan 13;13:1065598. doi: 10.3389/fpsyg.2022.1065598 (PMC9880425; doi:10.3389/fpsyg.2022.1065598)
Supplement: Supplementary file 1 [file Data_Sheet_1.docx]

|  | **NT**  **(*n* = 25)**  ***M* (SD)** | **ASD**  **(*n* = 29)**  ***M* (SD)** | **ADHD**  **(*n* = 27)**  ***M* (SD)** | **ASD+ADHD (*n =* 16)**  ***M* (SD)** |
| --- | --- | --- | --- | --- |
| Gender | 22 M; 3 F | 27 M; 2 F | 20 M; 7 F | 13 M; 3 F |
| Ethnicity: Hispanic *N* (%) | 1 (4.00) | 1 (3.40) | 4 (14.80) | 2 (12.50) |
| Race *N* (%) |  |  |  |  |
| White | 21 (84.00) | 24 (82.8) | 18 (66.70) | 13 (81.30) |
| >1 race | 1 (4.00) | 3 (10.3) | 8 (29.60) | 1 (4.00) |
| Asian | 1 (4.00) | 1 (4.00) | 0 (0.00) | 0 (0.00) |
| Black/African American | 1 (4.00) | 1 (4.00) | 0 (0.00) | 0 (0.00) |
| Primary Caregiver Bachelor’s Degree *N* (%) | 20 (80.00) | 13 (44.80) | 12 (44.40) | 5 (31.30) |
| Age in years | 8.30 (1.34) | 8.93 (1.30) | 8.50 (1.06) | 8.75 (1.34) |
| Full Scale IQ* | 118.96 (12.10) | 106.48 (12.13) | 115.70 (12.85) | 104.75 (12.60) |
| CBCL ADHD Symptoms Scale T-Score** | 51.48 (5.83) | 56.83 (3.79) | 69.50 (8.00) | 70.75 (3.84) |
| Social Responsiveness Scale Total T-Score*** | 55.39 (5.18) | 67.45 (9.73) | 61.33 (4.93) | 72.87 (7.68) |

**Supplementary Table 1.** *Demographic Characteristics. Note:* * Indicates TD, ADHD > ASD, ASD+ADHD at *p*s < .01; ** Indicates ADHD, ASD+ADHD > ASD > TD at *p*s < .001. ***Indicates ASD, ASD+ADHD > ADHD > TD at *p*s < .01

**
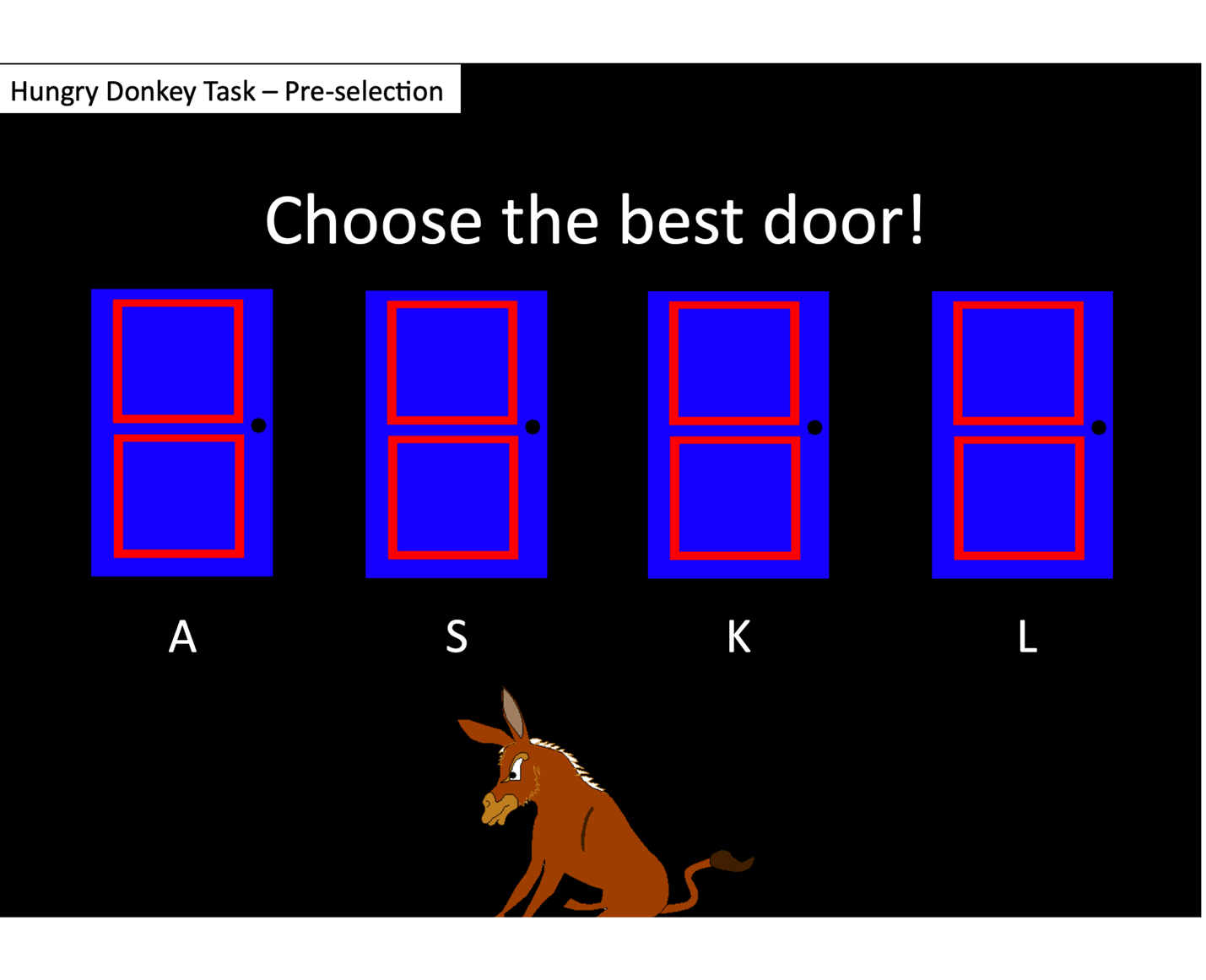
Supplementary Figure 1.** *Hungry Donkey Task, Pre-Selection. Note:* Task created in ePrime by Kiki Zanolie (Leiden University). Hungry Donkey Stimuli created by Crone & van der Molen, 2004.
